# Learning and Long-Term Retention of Large-Scale Artificial Languages

**DOI:** 10.1371/journal.pone.0052500

**Published:** 2013-01-02

**Authors:** Michael C. Frank, Joshua B. Tenenbaum, Edward Gibson

**Affiliations:** 1 Department of Psychology, Stanford University, Palo Alto, California, United States of America; 2 Department of Brain and Cognitive Sciences, Massachusetts Institute of Technology, Cambridge, Massachusetts, United States of America; UNLV, United States of America

## Abstract

Recovering discrete words from continuous speech is one of the first challenges facing language learners. Infants and adults can make use of the statistical structure of utterances to learn the forms of words from unsegmented input, suggesting that this ability may be useful for bootstrapping language-specific cues to segmentation. It is unknown, however, whether performance shown in small-scale laboratory demonstrations of “statistical learning” can scale up to allow learning of the lexicons of natural languages, which are orders of magnitude larger. Artificial language experiments with adults can be used to test whether the mechanisms of statistical learning are in principle scalable to larger lexicons. We report data from a large-scale learning experiment that demonstrates that adults can learn words from unsegmented input in much larger languages than previously documented and that they retain the words they learn for years. These results suggest that statistical word segmentation could be scalable to the challenges of lexical acquisition in natural language learning.

## Introduction

Spoken speech is a continuous acoustic waveform without consistent breaks at the boundaries between words. Although acoustic, phonetic, and prosodic features give partial evidence for where words begin and end, these cues vary widely between languages [1]. One source of information that is consistent across languages, however, is the statistical structure of the utterance itself [2]. Because utterances are generated by combining words from a finite lexicon, some sound sequences will be much more likely to appear than others. Hence, a learner can in principle work backwards from the distribution of sound sequences in a corpus of utterances to make an informed guess about the generating lexicon.

A variety of computational systems are now able to recover word boundaries with relative accuracy from an unsegmented corpus [3], [4], and laboratory experiments show that–at least under certain conditions–human learners can do the same thing. These experimental demonstrations (often referred to as “statistical learning” experiments) have used artificial languages with no prosody to show that both infants and adults are able to use the distribution of sound sequences to extract words from continuous speech [5], [6]. In a typical statistical learning experiment, infants or adults listen to a stream of unsegmented speech, generated by randomly concatenating words from a language containing 4–6 different word forms. After a very short exposure–sometimes as little as 2 minutes–listeners are then able to distinguish frequent sequences from less frequent distractors [5]. Infants in this type of experiment can even distinguish between strings that are matched for overall frequency but vary in their statistical coherence on measures like transitional probability (the probability of one syllable given the observation of another) [7].

What is the role that this kind of statistical learning plays in children's language acquisition? Some authors have suggested that it is an important part of the broader process of language acquisition [8]–[10], but others have questioned whether performance shown in short lab studies can scale up to the challenges of lexical acquisition [11]–[13]. In particular, it is unknown whether a mechanism that has only been demonstrated to operate over highly restricted artificial languages with homogeneous lexicons can nevertheless be applied successfully to the complex and heterogeneous lexicons of natural languages.

Recent work has found that learners can map meanings to the outputs of statistical segmentation tasks [14]–[16] and that statistical learning effects can be found using natural language stimuli [17], [18]. In addition, statistical learning effects are robust to variation in word and sentence lengths [19] and to Zipfian frequency distributions (the “heavy-tailed” distributions that are ubiquitous in natural languages, in which a few words appear with very high frequency while many others appear much more rarely [20]). But although the results of these tests have been positive, they do not fully address concerns regarding whether statistical learning can scale to larger languages and longer retention intervals, because they still use small-scale experimental tasks.

The goal of the current study is to address this concern about the scalability of statistical learning. We used adult learners to address this question, for two reasons. First, statistical learning abilities generally appear to be conserved across development [5], [6], [21], making adults a viable population for studying these abilities using large-scale and psychophysical paradigms not suited for infants and children. Second, although children learning new languages eventually reach higher levels of performance on complex syntactic and morphological regularities [22], they do not learn words faster or better than adults. In fact, memory for new lexical items increases considerably across development [23], [24], consistent with the increasing rate of vocabulary growth over the course of language acquisition [25], [26] and with general processes of maturation [27]. Our previous work has suggested that the major bottleneck in statistical learning tasks is memory for individual lexical items [19]. Thus, if adults are unable to learn words from a particular language via statistical learning, this failure should place an upper bound on children's abilities as well. Nevertheless, we note that a success by adults in learning a scaled-up language does not imply that statistical learning is used by children–only that negative arguments regarding scalability are invalid. Our current study was designed to evaluate these negative arguments.

In our study, four individuals listened to large corpora of synthesized speech, each over the course of a continuous ten-day period. Each participant listened for an hour a day on their iPod while they exercised, commuted to work, or relaxed, with the constraint that they did not read, speak, or otherwise use language during listening. The unique language that each participant heard was comprised of 1000 different words, which had the characteristic Zipfian frequency distribution of natural language, such that a few words were highly frequent while most others appeared only occasionally. The lengths of words and sentences were Poisson distributed, also as in natural language. Words were concatenated randomly without immediate repetitions so there was no syntactic structure available, but all sentences had a minimum of two words and a mean of four. Each of these factors has been studied in isolation [19], [20]; our intention here was to combine them on a much larger scale than previously attempted.

Because we wanted to test the scalability of statistical learning mechanisms, we chose to stay close in our paradigm to the original artificial language design pioneered by Saffran and colleagues [6], rather than adding additional cues like prosody [28]. In addition, because of the scope of our project, the use of natural language stimuli (as in [18]) would have been quite difficult. As a consequence, the only information that was present in our language but not in the initial experiments came from the boundaries between utterances. Although utterance boundaries are not necessary for learning (as shown by [6]), they are a pervasive feature of natural language, and our own previous data show that they facilitate segmentation performance [19]. Different accounts of segmentation treat utterance boundaries differently: while some treat them as merely another aspect of distributional structure (e.g., [3]), others have given them special status (e.g., [29]). For our purposes here we include these boundaries but note that they likely serve to make our languages easier to learn–though also more natural–than they would have been otherwise.

## Materials and Methods

In order to obtain a group of participants who would have a commitment to this relatively demanding experiment, we recruited from the population of research assistants in the MIT Brain and Cognitive Sciences Department. All participants gave written consent to participate in this research, and the details of this consent procedure were approved by the MIT Committee on the Use of Humans as Experimental Subjects. The final sample for the learning study consisted of four naïve members of the Brain and Cognitive Sciences community (1 MIT undergraduate, 1 student at another local institution, and 2 employees). They were matched with four yoked control participants. After three years, three of the four participants in the experimental condition were located for followup testing. One additional participant (a fifth) was excluded for using an explicit strategy during the initial test phase (placing a segment boundary every two syllables without variation throughout the entire test, rendering the initial test data uninterpretable).

A unique artificial language was generated for each participant. Each language had 1000 word types and 60,000 word tokens (for 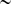
10 hours of speech). Frequencies of words were distributed via a Zipfian frequency distribution: 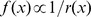, where 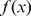 is the frequency of word 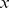 and 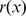 is its rank, such that there were a few highly frequent words and many more with lower frequencies (max = 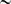
8000, min  = 10 tokens) [30]. Word lengths (in syllables) were generated by drawing from a Poisson distribution with mean 2 and adding 1 to avoid lengths of zero (mean = 3). The length and frequency of individual words were chosen independently: There was no bias to choose short words to be the highest frequency words in a language.

Words were created by combining 24 consonants and 14 vowels into 336 CV syllables and concatenating randomly. Sentences were then created by randomly concatenating words according to the frequency distribution of word types, with no word repeated immediately after itself (as in the initial work on statistical learning, which imposed this constraint to avoid the extra salience given by immediate repetitions [5], [6]). Following our previous work [19], [20], we synthesized our languages as a sequence of sentences. Sentences were distinguished from one another via a short but highly perceptible (200 ms) silence between them. Sentence lengths (in words) were generated by drawing from a Poisson with mean 2 and adding 2 to avoid sentences of length 1; the mean sentence length was 4 words (hence, 12 syllables).

Each training sentence was synthesized with no prosodic variations and no word boundaries using the MBROLA speech synthesis package with the us3 diphone database, with a duration of 250 ms per syllable and a constant F0 of 100 Hz [31]. The synthesizer was provided with unsegmented sequences and hence produced no temporal or coarticulation boundaries to distinguish between word-internal syllable transitions and word boundaries. Test materials were synthesized with the same settings.

Materials were generated as a series of 5 minute WAV files and loaded directly onto participants' personal iPod music players. Participants then listened to their language over headphones for approximately one hour each day over 10 days. They were instructed that they did not need to pay attention while listening but could not read, talk, or otherwise use language during the experiment; instead they were encouraged to listen while exercising or walking from place to place. To improve compliance, participants kept journals of listening activity; responses varied but the modal activities during listening were transportation and exercise.

Because two-alternative forced choice (2 AFC) trials impart information to participants about what the correct answers are (e.g. one of the two possibilities), it is not possible to conduct multiple testing sessions using a 2 AFC paradigm. To probe performance immediately after training [20], [32], we used an orthographic segmentation paradigm that tested participants' performance in making explicit word segmentation decisions In the first interim test session (“immediate test”), which occurred the day after they finished listening (the 11th day of the experiment), participants were tested on their ability to segment 400 tokens (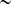
100 novel sentences). Orthographically glossed sentences–sentences written out as a string of syllables, as in “go lah bu pa doh ti”–were presented on a computer screen; participants were instructed to listen to the sentence as many times as they wanted and to click between syllables where they thought there was a break between words. Each of the four yoked control participants completed the same initial test as one participant in the study, but without completing the training session.

**Figure 1:**
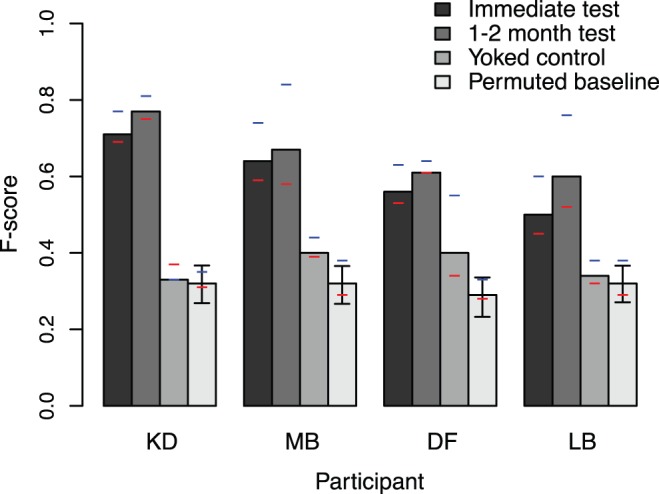
Results of the interim tests. Bars show F-scores (the harmonic mean of precision and recall) for the immediate and 1–2 month test sessions, along with permuted baseline and yoked control scores. Blue and red lines give precision and recall scores respectively for each participant and condition (means for permuted baseline). Error bars show 95% confidence intervals.

**Figure 3:**
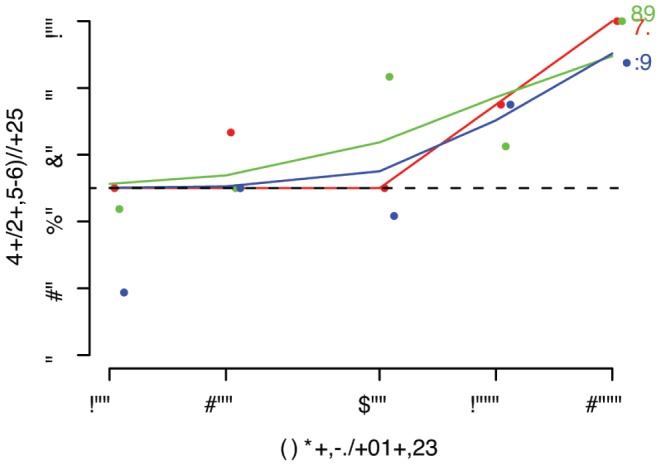
Percent correct performance on a set of 2 AFC test trials administered three years after training. Dots show individual participants' performance in one frequency range and are jittered slightly on the horizontal to avoid overplotting. Lines show best fitting half-logit regression models for individual participants.

The second interim test (“1–2 month test”) was identical to the first and was administered after one month (3 participants) or 2 months (1 participant, labeled LB in Figures 1 and 3). Participants had no further exposure to the corpus after the initial 10 day training session.

To provide measures comparable to those collected in previous work on statistical learning, the final test was a 2 AFC, administered approximately 3 years after the initial testing session (36–37 months). Participants listened to 64 MP3 files of pairs of words, synthesized as above. They were informed that one word was from the lexicon of the language they had initially heard during training, and that their job was to choose that word. Target words were sampled uniformly across the log frequency range spanned by the training sample, but all words above frequency 1000 were tested. Distractors were frequency-matched words from the lexicon of another participants' language (and contained syllables that were present in both languages). In order to avoid incentives for explicit study, participants were not notified that there would be a second interim test or a final test until several days beforehand, when they were contacted for scheduling.

## Results

All participants were able to segment novel sentences into their component words. Following the methods commonly used to evaluate computational studies of segmentation [3], [33], we compared participants' responses to the correct segmentation and computed *precision*, *recall*, and *F-score*. Precision and recall are signal detection-based measures that allow a set of responses to be evaluated independently from the decision threshold that is used. In our study, a “hit” was when a participant marked a boundary at a location where one existed, a “miss” was when a boundary was not marked by the participant, and a “false alarm” was when the participant marked a boundary in a location where there was not one. Precision was defined as hits/(hits+false alarms): the proportion of reported segmentation decisions that were correct. Recall was defined as hits/(hits+misses): the proportion of all correct segmentation decisions that were reported by the participants. It is common in the literature on computational linguistics to combine these two numbers for easy comparison by taking their harmonic mean, giving an F-score, a single number that is easily compared across conditions. Figure 1 shows these measures, both immediately after exposure and in a surprise 1–2 month followup test session.

Performance was relatively high, with F-scores generally above .5 and precision and recall relatively close to one another. Precision was higher than recall in all cases, suggesting that participants placed fewer boundaries than was appropriate, but that the boundaries they did place were accurate (in some cases over 80% correct). In addition, performance increased slightly from the first test to the 1–2 month followup. Although our small sample precludes making any inferences on the basis of this numerical increase, it could be due to a potential memory consolidation effect [34]. Alternatively, participants might have re-encoded the training materials during and after the first testing session, due to their presentation in the visual modality. In any case, we observed no decline in performance over the delay.

To create chance baselines for the F-score measure, we randomly permuted participants' own segmentation decisions. We created 10,000 simulated segmentations of each sentence for each participant: we took their initial segmentation of the sentence and shuffled the positions of the boundaries while keeping the number of boundaries constant. We then computed F-scores for each of these random segmentations and empirical 95% confidence intervals on these permuted F-scores. Using these baselines, we found that both immediately and 1–2 months later, participants performed considerably above chance (empirical 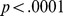). This result suggests that participants learned and retained the forms of the words and were able to apply this knowledge to make sensible decisions about how to segment speech in the language. In addition, because the baselines randomize individual participants' decisions within each sentence, they ensure that participants' accuracy was not due to guessing based on assumptions about the distribution of word lengths (as opposed to actual knowledge of word forms).

Performance was also well above the performance of the yoked controls, who received testing but no training. Although some of the yoked controls' performance was higher than baseline, even the most successful was still well below the performance of the least successful trained participant. This result suggests that performance in the initial segmentation task was not due to learning only the most frequent words (those that could be learned during the test session alone).

**Figure 2:**
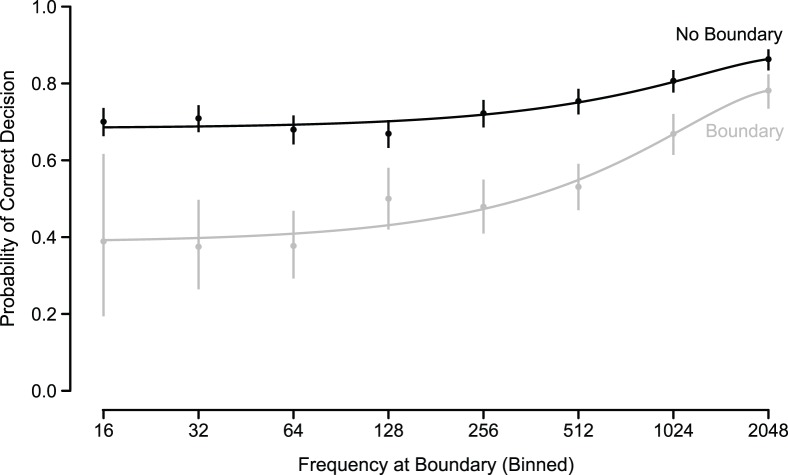
Probability of making a correct segmentation decision at a particular location in a sentence, plotted by whether there was a boundary at that location. Results are averaged across participants, and binned by the logarithm of the highest frequency word at the boundary (e.g., at the boundary between two words, the higher of the two word frequencies). Points show means, intervals show binomial 95% confidence intervals with a non-informative Beta prior, and lines show a loess smoother.

Further evidence that participants gained partial knowledge of many words–rather than learning just a few high frequency words–comes from an analysis of participants' boundary decisions at individual locations in sentences (Figure 2). We examined each decision on the basis of whether there was actually a word boundary at that location. Most words were longer than two syllables, so over all possible locations, more fell within words than between words. (If all words were two syllables, every other location would be a boundary, but since some words were three, four, or more syllables long, there were fewer boundaries than word-internal locations). Because participants were likely sensitive to this fact, there were more instances of correct rejections at word-internal locations, and overall performance on word-internal locations was higher than performance in finding boundaries.

To analyze the effects of frequency on segmentation performance, we classified decisions by the frequency of the word about which the decision was being made. For boundary locations, we used the higher frequency of the two words adjacent to the boundary. Overall, we saw a strong relationship between word frequency and segmentation performance. A linear mixed-effects model [35] confirmed this conclusion, finding effects of log frequency (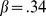, 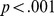), boundary presence (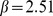, 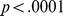), and their interaction (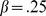, 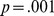). The formula used was corr.seg
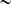
log(freq)* bound+(log(freq) * bound | subject), where corr.seg was an indicator variable for a correct segmentation decision, bound was an indicator variable for whether a boundary was present, and log(freq) was the natural logarithm of word frequency, described above. Significance was computed via the 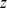-approximation due to the large number of observations and the relatively small anti-conservativity of this approach when a maximal random effects structure is used [36].

Three years after the initial experiment, we located three of four participants and administered a surprise test, asking them to distinguish words from novel length-matched distractors. A logistic mixed-effects model showed a highly significant effect of log frequency on performance (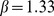, 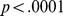), congruent with previous work on Zipfian frequency distributions showing that word frequency was the strongest predictor of accuracy at test [20]. Overall, while there was no evidence for retention of low-frequency words, retention of the high-frequency words was close to perfect despite the long period between training and test (Figure 3).

## Discussion

Our experiment was designed to test whether the abilities demonstrated in “statistical learning” tasks can be applied to large-scale lexicons. The evidence presented here suggests that they can. After ten days of exposure, learners acquired partial knowledge about many words in a massive artificial language, and retained the most frequent words across a three-year delay.

How does the scale of our experiment compare to natural language learning? Children hear 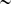
250,000–1,000,000 word tokens per month, for a total of 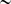
3–12 million words by their first birthday. If these tokens are produced in a Zipfian distribution over 20,000–60,000 word types, then the most frequent word will then be heard around 250,000–3,000,000 times, and the hundredth most frequent will still be heard several thousand times. (Sources for these figures: Hart and Risley [37] give an input range of 10–35 million words by age 3. The Human Speechome Corpus [38] contains approximately 16 million words in 15 months, for 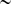
1 million words per month, again 36 million words by age 3. Average English vocabulary is around 60,000 words [39], though this may be significantly limited in child-directed speech.) Thus our data provide an in-principle demonstration that ambiguous contexts can lead to learning within both a frequency range and a retention interval comparable to natural language learning. Nevertheless, developmental experiments will be necessary to test whether statistical learning is a viable route to large-scale word learning for infants and children.

Exposure frequency (the number of times a string of sounds was heard) was the primary determinant of retention in our data. Previous work on word segmentation has suggested that learners succeed in statistical learning tasks by computing transitional probabilities (the probability 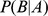 that some syllable 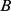 follows syllable 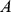 [5], [6]). Nevertheless the experimental data from statistical learning experiments are consistent with many possible psychological mechanisms, not just the transition probability computation [19], [20], [40]. One class of “chunking” models relies on memory mechanisms to extract and retain an internally-consistent segmentation of the input into frequent chunks [3], [41], [42]. Chunking models that have interference effects or parsimony biases could provide a good explanation for the frequency dependence of learners' performance, while also capturing transitional probability effects. Thus, “frequency or transitional probability” may be the wrong question. Instead, future research should investigate proposed mechanisms that capture both smaller-scale transitional probability effects and large-scale frequency dependence.

Although our experiments were not directly designed to test the connection between memory mechanisms and statistical learning, there are nevertheless similarities between our results and several studies of language learning and long-term memory. First, the dependence of performance on log word frequency parallels the relationship found by Anderson [43] and others. Second, the scale of learning is consistent with previous work on long-term lexical memory [44]. Third, many models of language learning assume that only the highest-frequency forms are retained and used for inferences [45], [46]. Finally, although comparable studies have not been performed, children's retention of novel word forms and meanings over intervals of weeks or months has been well-documented [47], [48].

Despite limited experimental evidence, the utility of exposure to language input without direct interaction–via television, radio, podcast, or overheard speech–is widely debated in informal discussions of second language learning. Our results show that for adults, this kind of exposure can promote the long-term retention of high-frequency, statistically-coherent chunks of language, albeit without any links to meaning. This kind of exposure may create a baseline competence for future comprehension in meaningful settings, useful both for prelinguistic infants who hear large amounts of speech before they begin producing or comprehending language and for adults learning to parse an unfamiliar language.
