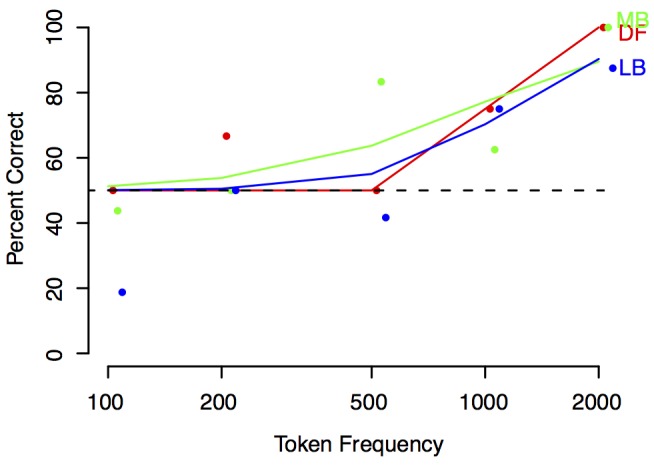# Correction: Learning and Long-Term Retention of Large-Scale Artificial Languages

**DOI:** 10.1371/annotation/fcf19f94-5f2c-4a5f-9a6f-3d880bcdd850

**Published:** 2013-04-11

**Authors:** Michael C. Frank, Joshua B. Tenenbaum, Edward Gibson

The version of Figure 3 displays its text incorrectly. There correct version is available here: 

**Figure:**